# Development and validation of a questionnaire to measure COVID-19 vaccine hesitancy within the Romanian industrial laborers

**DOI:** 10.3389/fpubh.2025.1482778

**Published:** 2025-02-27

**Authors:** Adina Turcu-Stiolica, Ancuta Ramona Boicea, Florina Nechita, Roxana Surugiu, Gheorghe Gindrovel Dumitra, Carmen Nicoleta Oancea, Mihail Cristian Pîrlog

**Affiliations:** ^1^Department of Pharmacoeconomics, University of Medicine and Pharmacy of Craiova, Craiova, Romania; ^2^Department of Occupational Medicine, University of Medicine, and Pharmacy of Craiova, Craiova, Romania; ^3^Department of Medical Psychology, University of Medicine, and Pharmacy of Craiova, Craiova, Romania; ^4^Department of Biochemistry, University of Medicine, and Pharmacy of Craiova, Craiova, Romania; ^5^Department of Family Medicine, University of Medicine, and Pharmacy of Craiova, Craiova, Romania; ^6^Department of Medical Sociology, University of Medicine, and Pharmacy of Craiova, Craiova, Romania

**Keywords:** COVID-19, COVID-19 vaccine, vaccine hesitancy, Romania, questionnaire, COVID-19 vaccination

## Abstract

The identification and quantification of sources of vaccine hesitancy among industrial workers in Romania have become crucial for developing effective strategies to facilitate the vaccination process. Our study included employees, both with and without comorbidities, who work in industrial companies. The goal was to develop a scale to assess COVID-19 vaccine hesitancy in Romania. This proposed scale has been designated as the Romanian COVID-19 Vaccine Hesitancy (RO-CVH) scale. The survey encompassed both the demographic characteristics of the respondents and questions related to their perceptions of COVID-19 vaccination. A three-stage process was used to develop the RO-CVH which includes (1) item generation; (2) item-refinement (pilot testing, exploratory factor analysis); and (3) scale validation. The fifteen items loaded onto three factors using exploratory factor analysis, explaining 63% of the total variance. The three factors were labelled as “Confidence in information regarding the COVID-19 vaccine,” “Safety and efficacy of the COVID-19 vaccine,” and “COVID-19 vaccination as a means of controlling the population.” The content validity of the scale was established, and it will be utilized to comprehend the behavior of industrial workers in Romania during similar future outbreaks, particularly regarding the acceptance of mitigatory vaccines. Based on the insights from this scale, future interventions could be designed to reduce vaccine hesitancy.

## 1 Introduction

At the end of 2020, the COVID-19 pandemic was estimated to have reduced the world’s collective gross domestic product (GDP) by 3.4 percent, equivalent to more than US$2 trillion (1). Current studies suggest that by the end of 2023, the economic impact of the pandemic in the United States alone will reach US$14 trillion (2). A substantial portion of this economic toll stems from the challenges faced by various sectors, including industry and manufacturing.

Romania has encountered multiple successive waves of the COVID-19 pandemic commencing in 2020. The fourth wave, which transpired during the autumn of 2021, was predominantly driven by the Delta variant, thereby resulting in a substantial surge in the incidence of COVID-19 cases, hospital admissions, and fatalities (3). Romania’s vaccination campaign was launched in December 2020, initially focusing on the deployment of mRNA-based vaccines and prioritizing healthcare professionals in hospital environments (4).

The period leading up to May 2021 witnessed the vaccination campaign operating under the constraints imposed by vaccine accessibility. As the pool of individuals voluntarily seeking vaccination diminished, authorities were confronted with the challenges posed by vaccine hesitancy as a prevailing phenomenon. Consequently, targeted interventions became imperative, particularly within specific demographic cohorts exhibiting resistance or hesitancy toward vaccination (4). By September 2021, a total of 5,142,278 individuals in Romania had completed the vaccination regimen with mRNA and viral vector-based vaccines, representing approximately 27.9% of the general population. This low vaccination coverage positioned Romania second to last among European Union countries (5).

Vaccine hesitancy was classified as one of the top 10 global health threats by the World Health Organization (WHO) (6). This phenomenon arose in the context of a broad decline in trust in vaccines, influenced by factors such as the growing impact of the anti-vaccine movement and the reduced visibility of vaccine-preventable diseases. Paradoxically, the success of vaccines has contributed to their own undoing, as some previously eradicated diseases now face the potential of reemergence (7, 8).

Unpublished data from various organizations, including the WHO, in Romania, have highlighted that family doctors and employers are held in higher regard in terms of trust. Consequently, it became apparent that individualized counseling and group facilitation interventions, carried out in the workplace by family doctors and company physicians, could be particularly effective, especially in industrial units where employees from both rural and urban areas converge. When it comes to measure vaccination hesitancy, several methods have been proposed especially for gauging parental hesitancy to vaccinate their children. They were used in the pre-pandemic period (9, 10). Vaccinating adults presents unique challenges, particularly when introducing new vaccines that utilize innovative technologies, all within a context of widespread misinformation surrounding vaccines.

Given the above-mentioned substantial economic costs of the pandemic and its profound impact on industrial production, notably constrained by lockdown measures, the vaccination process has been regarded as one of the vital lifelines required for the sustainable recovery of this economic sector (11). Therefore, it is crucial to identify and quantify the sources of vaccine hesitancy among industrial workers, with the goal of developing strategies to facilitate a sustainable vaccination process. This has become the primary objective of our research, focusing on the validation of a measurement instrument specifically designed to assess vaccine hesitancy within this population group. Future studies will utilize this instrument to develop targeted interventions aimed at addressing vaccine hesitancy and promoting vaccine uptake among Romanian industrial laborers.

## 2 Materials and methods

The development of the new tool began with the careful formulation of items, ensuring their content validity. The items were developed through a combination of methods, including a comprehensive review of relevant literature, and several rounds of content validity evaluations conducted by experts. This was followed by a thorough scientific examination, which included exploratory factor analysis and an extensive assessment of the instrument’s validity, supported by existing research (12, 13). It is worth noting that the study in question received ethical approval from the Ethics Committee of the University of Medicine and Pharmacy of Craiova, Romania (no. 174/29.10.2021).

### 2.1 Questionnaire development – item generation

In an effort to glean deeper insights from participants, a COVID-19 vaccine hesitancy questionnaire consisting of 17 items was crafted, guided by existing literature and meticulously analyzed by two experts (G.D. and M.P.). These items were carefully formulated to eliminate ambiguity and were presented in a format devoid of loaded language. To gauge hesitancy toward COVID-19 vaccination, participants rated their responses on a 5-point Likert scale: 1 = ‘Strongly disagree’, 2 = ‘Disagree’, 3 = ‘Neutral’, 4 = ‘Agree’, and 5 = ‘Strongly agree’.

To assess the initial validity of the questionnaire, a preliminary pilot study was conducted involving 30 participants. This phase aimed to refine the instrument, necessitating revisions in language, removal of ambiguous terms, and elimination of technical jargon. These refinements were essential in ensuring the questionnaire’s clarity and appropriateness for subsequent research use.

### 2.2 Questionnaire development – data collection

Participants eligible for inclusion were those aged 18 to 65 years, with the cognitive ability to comprehend and make informed decisions regarding their health. The study included individuals across all educational backgrounds, from primary and secondary education to higher education.

The selection process was carried out randomly by the occupational medicine physician within industrial units where employer consent had been obtained.

Employees who had already received a COVID-19 vaccine were excluded from the study. The dataset exhibited a complete absence of missing values.

### 2.3 Questionnaire development – evaluation

The Kaiser-Meyer-Olkin (KMO) criterion test was employed to assess the adequacy of the sample size for a stable factor solution (14). Determinant and Bartlett’s test of sphericity were utilized to confirm the stability of the factors and ascertain if the items exhibited sufficient interrelatedness to conduct a meaningful Exploratory Factor Analysis (EFA) (15). In the EFA, extraction was based on Eigenvalues greater than 1, ensuring maximum variance explanation and grouping items based on strong correlations. Two rotation methods were employed to enhance factor differentiation: oblique and varimax.

Face validity was confirmed to validate the relevance of the extracted factors and assess if the items loaded cohesively on the same factor.

Reliability was assessed by computing Cronbach’s alpha for each factor. A value of *α* > 0.70 was deemed preferable for constructing the new scale.

The COVID-19 vaccine hesitancy score was calculated as the sum of all scored items divided by the total number of final items. Similarly, scores for individual factors were computed using the same approach.

Validity was evaluated using a Visual Analogue Scale (VAS), ranging from 0 to 10, where 0 indicated ‘No COVID-19 vaccination willingness’ and 10 represented ‘Full COVID-19 vaccination willingness’. Spearman correlation coefficients were calculated to assess the correlation between questionnaire scores and the VAS score (VScore).

### 2.4 Statistical analysis

All analysis were performed in GraphPad Prism 9.4.1 (GraphPad Software, San Diego, CA, United States). Descriptive analysis was done for continuous (mean, standard deviation) and categorical (frequencies, percentages) variables. Prior to EFA, KMO and Bartlett’s Test of Sphericity were determined. Cattell’s scree plot was used to visual display elbow which indicates the cutoff point for factor extraction.

We graphed the heatmap of the correlation matrix, after computing nonparametric Spearman correlation two-tailed. Color mapping ranged from green for the largest value to magenta for the smallest value, each cell being labeled with its value. A *p*-value less than 0.05 was considered statistically significant.

## 3 Results

In the pilot phase, the initial paper-based questionnaire comprising 20 questions was administered to 30 participants to evaluate the clarity of the items. In the process, three questions employing binary yes/no responses were excluded from the section where the COVID-19 vaccine hesitancy score was calculated. Additionally, ten items formulated in a negative manner were reverse coded to ensure consistency in responses. The participants took between 5 to 10 min to complete the final questionnaire.

### 3.1 Sociodemographic characteristics

In the study, a total of 256 participants were included, with 69.1% being male. The sociodemographic details of the participants are outlined in Table 1. The average age of the participants was 41.3 years, ranging from 19 to 64 years. A significant proportion of the participants (70.3%) had an education level of high school or below. Moreover, the majority of participants (64.1%) resided in urban areas. Additionally, 71.9% of the participants were married, and 70.7% had children.

**Table 1:** Socio-demographic characteristics of the participants to the intervention.

Characteristics	Total (256 participants) *n* (%)
Age (years), mean ± SD	41.3 ± 11.1
Gender	
Females	79 (30.9%)
Males	177 (69.1%)
Marital status	
Married	184 (71.9%)
Singles/divorced	72 (28.1%)
Educational level	
Elementary school	18 (7.0%)
High school	162 (63.3%)
University degree	55 (21.5%)
Master/PhD	21 (8.2%)
Residence place	
Urban	164 (64.1%)
Rural	92 (35.9%)
Having children, yes	181 (70.7%)
Having chronic disease, yes	46 (18%)
Previous COVID-19 diagnosis, yes	47 (18.4%)
Annual flu vaccine, yes	36 (14.1%)
Anti-vaccination beliefs in general, yes	44 (17.2%)

^1SD, standard deviation.^

The respondents had relatives or friends that were diagnosticated with COVID-19 (*n* = 132, 51.6%) and, within them, 18.9% (*n* = 25) had severe COVID-19 or died from COVID-19. Also, only a percentage of 26.6% (*n* = 68) believed COVID-19 had natural source from animals, whereas the rest of the participants (*n* = 188, 73.4%) believed COVID-19 was created by humans.

### 3.2 Exploratory factor analysis

**Figure 1:**
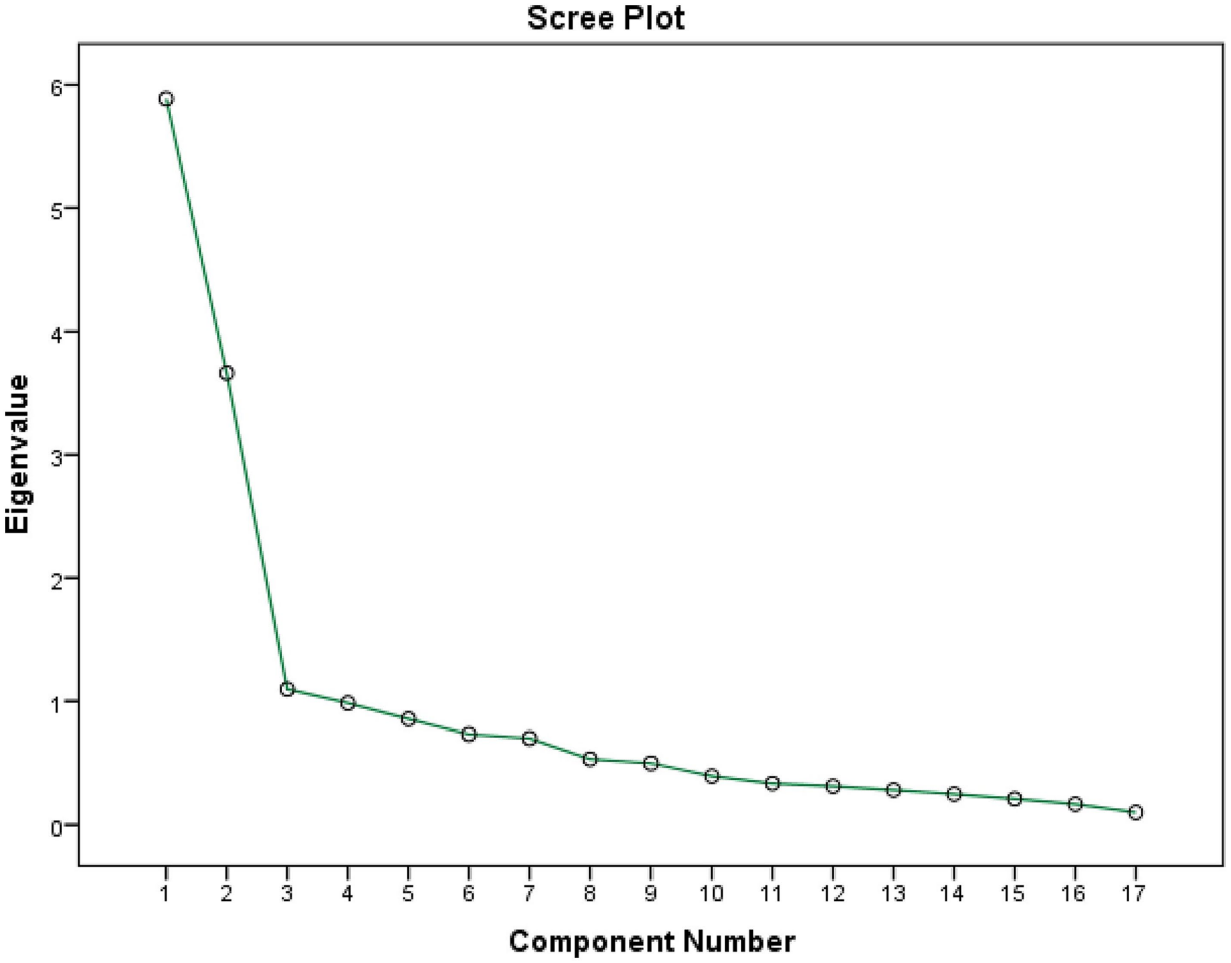
Scree plot of exploratory factor analysis.

The value of Kaiser-Meyer-Olkin (KMO) measure of sampling adequacy (KMO) is greater than 0.5 (KMO = 0.878), demonstrating we did not have sample size issue. The result of the test of sphericity from Bartlett demonstrated we had at least one significant correlation between two of our items (*p* < 0.001), so we had a meaningful exploratory factor analysis. The total variance of the 17 items explained by the 3 factors was 62.62% (Figure 1; see Table 2).

**Table 2:** Shows the factor loadings of the 17 items to the three factors extracted (bold questions were excluded from the final questionnaire).

Item	Factors		
	1	2	3
	Confidence in information regarding the COVID-19 vaccine	Safety and efficacy of the COVID-19 vaccine	COVID-19 vaccination is a way of controlling the people
Q1. I believe that the COVID-19 vaccine is a way to control people.			0.550
**Q2. I believe that the COVID-19 vaccine leads to infertility.**		0.476	
	
Q3. I do not consider it acceptable to impose the anti-COVID-19 vaccination.			0.771
Q4. Data on vaccination safety are often false.	0.640		
Q5. I do not think the COVID-19 vaccine is safe.		0.864	
Q6. I do not think it’s important to get the COVID-19 vaccine to protect ourselves.		0.896	
Q7. I do not think it’s important to get the COVID-19 vaccine to protect our family.		0.882	
Q8. I do not think it is important to make the COVID-19 vaccine to control the pandemic.		0.875	
Q9. Immunizing children is harmful, and this is hidden by not being told.	0.760		
Q10. Pharmaceutical companies are hiding the side effects of the COVID-19 vaccines.	0.788		
Q11. Vaccines only serve pharmaceutical companies that want to get rich.	0.777		
Q12. I do not think the COVID-19 vaccine is effective.		0.876	
Q13. I do not think it’s important to get vaccinated against COVID-19 to get back to normal life.		0.880	
**Q14. COVID-19 vaccines are not consistent with my religious beliefs.**		0.592	
Q15. Data on the effectiveness of vaccination are often false.	0.756		
Q16. I am afraid of the adverse effects of the COVID-19 vaccine.	0.680		
Q17. The vaccine was not studied enough before it was approved for vaccinating people.	0.785		


We measured reliability within every factor. Cronbach’s alpha for factor 1 (Confidence in information regarding the COVID-19) was 0.867 and all the items remained included in the factor (no better values for Cronbach’s alpha were obtained if one item deleted). Cronbach’s alpha for factor 2 (Safety and efficacy of the COVID-19 vaccine) was 0.948 after we deleted item Q2 and Q14. Cronbach’s alpha for factor 3 (COVID-19 vaccination is a way of controlling the people) was 0.802 and all the items remained included in the factor (no better values for Cronbach’s alpha were obtained if one item deleted).

COVID-19 vaccine hesitancy score was calculated as the sum of all scored items divided by the number of items (12). In the same way, we calculated the score for every factor. We developed a self-report measure, Romanian COVID-19 Vaccine Hesitancy (RO-CVH), containing 15 items and intending to better understand individuals’ concerns about COVID-19 vaccines through 3 factors. In our sample, the Cronbach’s alpha factor for entire questionnaire with 15 final items was 0.842 and inter-item correlations ranged from 0.041 to 0.921, with the variance = 0.063.

### 3.3 Construct validity

**Figure 2:**
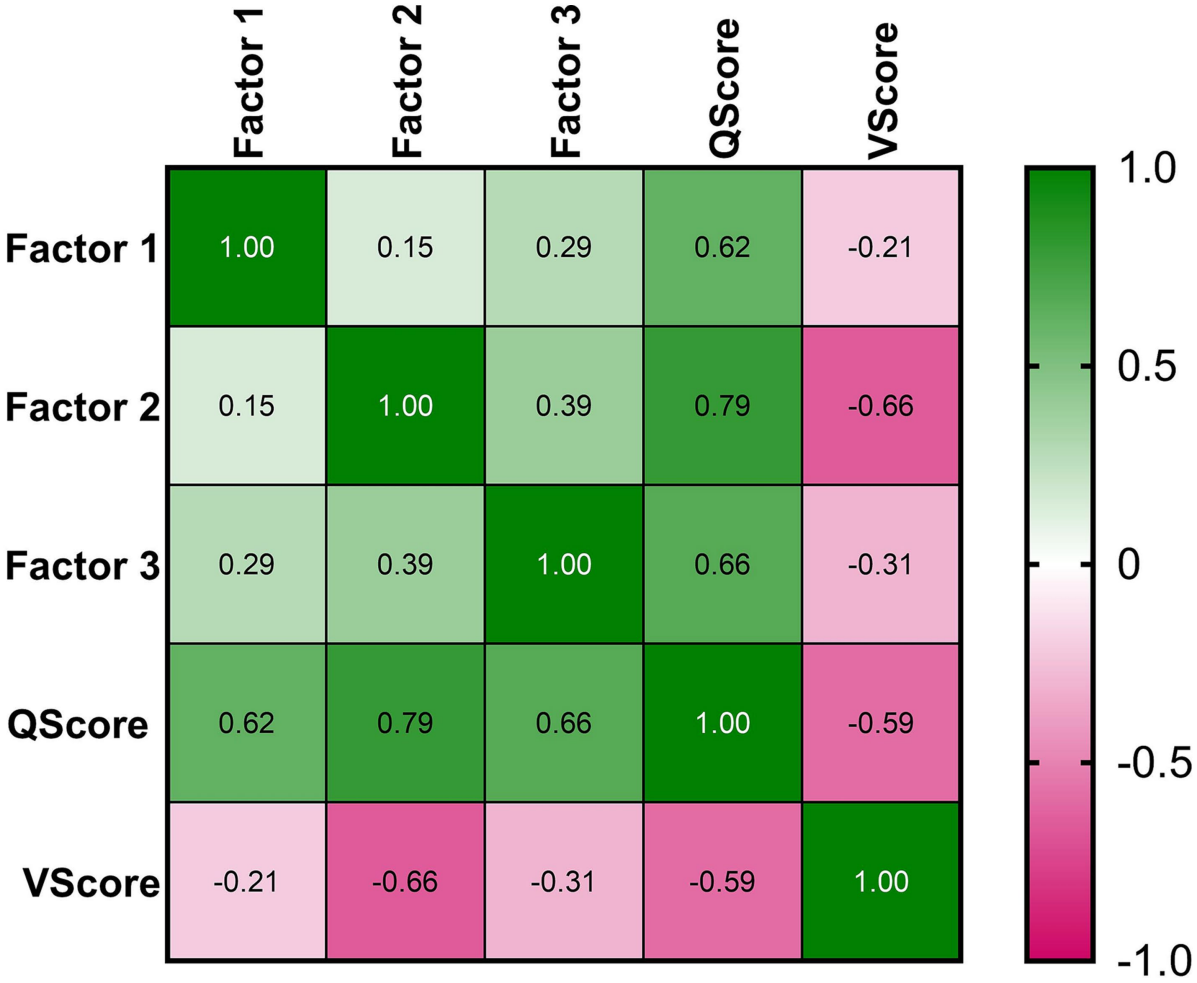
Heatmap correlation. The mapping ranged from green for the largest value to magenta for the smallest value, each cell being labeled with its Spearman coefficients values.

Significant correlations (*p* < 0.001) were found between the total score, the three factors and the VAS score, as in Figure 2. The score obtained from our questionnaire ranged between 21 and 93%, with mean ± SD of 56.7% ± 13.1%.

The moderate correlation (rho = −0.59, *p* < 0.001) between COVID-19 vaccination willingness (VScore) and COVID-19 vaccine hesitancy (QScore) indicates the validity of our questionnaire and vaccine hesitancy to predict COVID-19 vaccination status.

## 4 Discussion

This study aimed to create and validate a scale for evaluating concerns related to COVID-19 vaccines. The resultant scale RO-CVH is composed of 15 self-report items on a 5-point Likert scale. The score of RO-CVH describes the level 0f COVID-19 vaccine hesitancy, and significantly predicts COVID-19 vaccination status, assessing the factors influencing the decision for COVID-19 vaccination. Our scale offers a validated approach to understand what factors actively make the decision to receive or not the vaccine: confidence in information regarding the COVID-19 vaccine, safety and efficacy of the COVID-19 vaccine, or the belief that COVID-19 vaccination is a way of controlling the people.

To achieve effective disease control during a pandemic, attaining herd immunity is essential. This immunity can be acquired either through natural infection or through widespread immunization. However, relying on natural infection poses substantial risks, including high mortality rates, overwhelming healthcare systems, and significant disruptions to societal functions. Consequently, immunization emerges as the most viable and strategic approach to mitigating the impact of pandemics, ensuring both public health and societal stability (16). Of particular importance is the impact of illnesses among employees in industry, a phenomenon that causes absenteeism and reduced yields, a fact that has a dramatic impact on the economy and on the quality of life (17).

To obtain herd immunity, a large percentage of the population must be vaccinated. When the level of vaccine hesitancy is high, knowing the reasons behind it becomes very important (11, 16). The role of healthcare workers is pivotal in fostering and maintaining public trust (18).

While instruments exist for assessing knowledge, attitudes, and practices that contribute to adherence to infectious disease control guidelines in COVID-19 (19–21), there remains a critical need to identify and understand the factors driving vaccine hesitancy and reluctance specific for each populational group. Kricorian et al. conducted a nationwide survey in the United States to assess adults’ health literacy regarding the COVID-19 vaccine, as well as their beliefs and experiences related to the virus. However, it is important to note that the instrument used in this study was not fully validated (22). Similarly, other studies were identified that presented various problems regarding their validation (23–25) as well as the general field of application (26).

Our questionnaire demonstrates strong construct validity, offering valuable insights into the underlying factors associated with vaccine hesitancy among adults. Specifically, it focuses on hesitancy related to COVID-19 vaccination and gathers comprehensive data throughout the entire vaccination campaign. Notably, this questionnaire stands as one of the pioneering tools tailored for employees within the industry, making it unique in addressing the active adult population. Its significance lies in its potential to enhance our understanding of the imperative need for vaccination.

This instrument is characterized by its ease of administration, enabling the comparison of knowledge, behaviors, and attitudes among individuals residing in both urban and rural areas. This feature is particularly significant as researching trends within rural populations often presents challenges in identifying barriers (27). A survey conducted in rural Oklahoma found that COVID-19 vaccination hesitancy was significantly influenced by factors such as mistrust in the vaccine’s safety and efficacy, limited access to reliable health information, and strong community-held beliefs, highlighting the need for targeted interventions to address the unique challenges faced by this population (28). Consequently, our questionnaire not only fills a critical gap in the literature but also provides a valuable tool for exploring vaccination attitudes and behaviors, especially among adult populations actively engaged in various industries. Several studies highlight the significant impact of socioeconomic factors on vaccine uptake, even in the presence of detailed vaccination regime and free access to vaccines (29, 30). Also, when taking into account the size of the company a study in Japan identified that second-dose coverage was notably lower among employees of small companies, largely due to fewer employer-arranged vaccination opportunities and socioeconomic factors (31).

The RO-CVH scale provides a valuable tool for identifying and addressing the specific factors driving vaccine hesitancy, such as misinformation, trust in healthcare systems, perceived risks, and workplace culture, to design targeted vaccination campaigns tailored to address specific concerns. Beyond COVID-19, it can inform vaccination efforts for influenza and future outbreaks, ensuring sustainable improvements in vaccine acceptance. As part of our ongoing efforts, we have applied similar tools to investigate influenza vaccine refusal across various populations, considering the values and characteristics of our communities (32).

We acknowledge several limitations in our study. Firstly, the research sample was exclusively drawn from heavy industry entities, leading to a gender distribution disparity with a predominant male presence. Secondly, the rapid progression of vaccination stages, alongside the necessity for urgent professional information intervention within this specific population, restricted the possibility of retesting and comparing the initial results. While this study provides a validated tool to assess COVID-19 vaccine hesitancy among Romanian industrial laborers, it is important to note that the scale primarily focuses on individual-level factors influencing hesitancy, such as perceptions of vaccine safety, efficacy, and trust in health authorities. It does not extensively address systemic issues, including structural barriers to vaccine access (e.g., availability, affordability, and logistical challenges) or the pervasive impact of misinformation and disinformation campaigns. These systemic factors are critical in shaping vaccine hesitancy and uptake, particularly in low-resource or high-misinformation environments. Future research should aim to incorporate these dimensions to provide a more comprehensive understanding of vaccine hesitancy and inform targeted interventions. These constraints should be considered when interpreting the findings of our study.

## 5 Conclusion

Our questionnaire serves as a validated and reliable instrument designed to assess vaccine hesitancy among industry workers. This tool is invaluable for employers and medical services within the industry, enabling them to discern the factors contributing to low acceptance rates and vaccine refusals, not only during the COVID-19 pandemic but also in other contexts such as outbreaks or annual influenza vaccinations. By understanding the root causes of vaccine hesitancy, a crucial step in promoting sustainable health practices, tailored interventions can be developed. Optimal vaccination coverage can only be achieved through interventions precisely tailored to the identified causes of hesitancy. Having a validated questionnaire to pinpoint vaccine hesitancy equips us with a practical tool. It allows us to implement targeted interventions and design sustainable policies within industrial settings. These efforts, in turn, can significantly reduce workplace absenteeism, mitigate losses in qualified or highly skilled human resources, thereby positively impacting productivity levels.

## Data Availability

The original contributions presented in the study are included in the article/supplementary material, further inquiries can be directed to the corresponding authors.
